# Secretory Leukocyte Protease Inhibitor Is Present in Circulating and Tissue-Recruited Human Eosinophils and Regulates Their Migratory Function

**DOI:** 10.3389/fimmu.2021.737231

**Published:** 2022-01-12

**Authors:** Oktawia Osiecka, Joanna Skrzeczynska-Moncznik, Agnieszka Morytko, Angelika Mazur, Pawel Majewski, Bernadetta Bilska, Monika Kapinska-Mrowiecka, Joanna Kosalka-Wegiel, Maciej Pastuszczak, Elzbieta Pyza, Joanna Cichy

**Affiliations:** ^1^ Department of Immunology, Faculty of Biochemistry, Biophysics and Biotechnology, Jagiellonian University, Kraków, Poland; ^2^ Department of Cell Biology and Imaging, Institute of Zoology and Biomedical Research, Jagiellonian University, Kraków, Poland; ^3^ Department of Dermatology, Zeromski Hospital, Kraków, Poland; ^4^ II Department of Internal Medicine, Jagiellonian University Medical College, Kraków, Poland; ^5^ Department of Rheumatology and Immunology, University Hospital, Kraków, Poland; ^6^ Department of Dermatology, Jagiellonian University Medical College, Kraków, Poland

**Keywords:** secretory leukocyte protease inhibitor, granulocyte, eosinophil, eosinophilic granulomatosis with polyangiitis (EGPA), atopic dermatitis (AD)

## Abstract

Eosinophils and secretory leukocyte protease inhibitor (SLPI) are both associated with Th2 immune responses and allergic diseases, but whether the fact that they are both implicated in these conditions is pathophysiologically related remains unknown. Here we demonstrate that human eosinophils derived from normal individuals are one of the major sources of SLPI among circulating leukocytes. SLPI was found to be stored in the crystalline core of eosinophil granules, and its dislocation/rearrangement in the crystalline core likely resulted in changes in immunostaining for SLPI in these cells. High levels of SLPI were also detected in blood eosinophils from patients with allergy-associated diseases marked by eosinophilia. These include individuals with eosinophilic granulomatosis with polyangiitis (EGPA) and atopic dermatitis (AD), who were also found to have elevated SLPI levels in their plasma. In addition to the circulating eosinophils, diseased skin of AD patients also contained SLPI-positive eosinophils. Exogenous, recombinant SLPI increased numbers of migratory eosinophils and supported their chemotactic response to CCL11, one of the key chemokines that regulate eosinophil migratory cues. Together, these findings suggest a role for SLPI in controlling Th2 pathophysiologic processes *via* its impact on and/or from eosinophils.

## Introduction

Eosinophils are one of the principal cellular components of the host response to helminth infection and participate in several allergy-associated inflammatory diseases, such as eosinophilic granulomatosis with polyangiitis (EGPA) and atopic dermatitis (AD). They are also implicated in the host response to fungal, viral, and bacterial infections, tissue development and remodeling, metabolic homeostasis, and regulation of both innate and adaptive immunity, mainly through their cross talk with mast cells and plasma cells, respectively (1, 2). Eosinophils are evolutionarily maintained in all vertebrates. There are no reported cases of congenital eosinopenia in humans, whereas infection with parasites or allergic hypersensitivity is commonly associated with an often dramatic rise in blood or tissue eosinophil counts (1, 2). Although these data suggest that eosinophils are evolutionarily beneficial, specific functions of eosinophils in immunity remain elusive. This is partly due to the phenotypic and functional differences between human and mouse eosinophils, which limit the usage of eosinophil-deficient experimental models in order to delineate eosinophil contributions to human health and disease mechanistically (1).

Terminally differentiated eosinophils are released into circulation from the bone marrow and mobilized into tissues in response to specific chemoattractants. The members of the eotaxin family of chemokines, such as eotaxin-1 (CCL11), are among the main drivers of eosinophil tissue recruitment (1). Eosinophils are equipped with several unique features that likely play a key role in eosinophil responses to parasite infections and allergen challenge. These include distinctive cationic proteins, such as major basic protein (MBP), the principal constituent of the crystalline core, and eosinophil cationic protein (ECP), which are stored in eosinophil granules and can be released into the extracellular milieu through degranulation. Eosinophils can also serve as effector and regulatory immune cells by releasing inflammatory lipid and protein mediators' and mitochondrial DNA (1, 2).

One human condition marked by hypereosinophilia is EGPA (formerly known as the Churg–Strauss syndrome), (1). In EGPA, inflammation of the blood vessels can restrict blood flow to organs and tissues. Patients with EGPA have a history of eosinophilic asthma and sinusitis often associated with nasal polyps. It was found that biological therapy targeting IL-5, one of the key cytokines involved in eosinophil biology, resulted in a marked depletion in blood and sputum eosinophil counts and a modest (50%) reduction in bronchial wall eosinophils. IL-5-triggered eosinopenia was accompanied by significantly fewer exacerbations in patients with severe eosinophilic asthma (3, 4). The administration of an anti-IL-5 antibody also showed some effects on the clinical symptoms of relapsing or refractory EGPA. These include a significantly longer time in remission and a higher proportion of participants in remission compared to the placebo group. However, only approximately half the participants clinically responded to this therapy (5).

Peripheral blood eosinophilia is also a typical feature of AD, -chronic eczematous skin disorder. High-circulating or tissue eosinophil counts or eosinophil granule protein levels were linked to AD severity (6) and elevated IgE levels (7). However, anti-IL-5 strategies resulted in only moderate efficacy in AD treatment (8).

Although in many patients with eosinophilic tissue disease the cause remains obscure, the more or less robust beneficial clinical outcome of eosinophil-targeted therapy suggests the role of these cells in the pathogenicity of human allergy-related disorders. However, the somewhat unclear role of eosinophils in tissue dysfunction revealed by the modest clinical effects of eosinophil-targeted strategies in many patients requires further insights into eosinophil function and the regulation of eosinophil activity.

One of the molecules associated with eosinophilic asthma or AD-affected skin is the secretory leukocyte protease inhibitor (SLPI) (9–11). SLPI is equipped with a range of immunomodulatory activities, including anti-protease function and the inhibition of NFκB activities in an anti-protease-independent manner. Under inflammatory conditions, SLPI is thought to be generally protective by counteracting excessive inflammatory responses and supporting tissue healing (12).

Elevated levels of both eosinophils and SLPI in tissues affected by allergy-associated inflammatory diseases suggest that eosinophils may be a source and/or target of SLPI. This is in line with a recent study that demonstrated that SLPI is expressed in mouse eosinophils and that the ability of SLPI-deficient murine eosinophils to secrete IL-6 and express metalloproteinase-9 (MMP-9) is diminished (13). However, SLPI expression and/or function in human eosinophils remain unknown. Given that rodent and human eosinophils differ substantially (1), it is important to determine whether SLPI is involved in the biology of human eosinophils.

Here we report that human eosinophils contain one of the highest levels of SLPI among major blood leukocyte types. Both healthy individuals and patients with eosinophilia were uniformly found to contain SLPI-positive circulating eosinophils. Eosinophils infiltrating AD skin immunostained for SLPI, whereas exogenous recombinant SLPI increased the number of migratory eosinophils *in vitro*. Taken together, these findings suggest that SLPI regulates eosinophil ability to migrate, which may be relevant to eosinophil tissue recruitment under homeostatic and/or allergy-associated inflammatory conditions.

## Materials and Methods

### Materials

Recombinant SLPI was produced as previously described (14). Recombinant human eotaxin-1 (CCL11) was obtained from PeproTech (Rocky Hill, NJ, USA).

For SLPI staining, three monoclonal Abs were used: biotin-mouse-anti-human SLPI (clone: 31, Abcam); mouse-anti-human SLPI (clone 20409, Creative Diagnostics); mouse-anti-human SLPI (clone 20409, R&D); isotype control antibody included; biotin mouse IgG1 к (clone: MOPC-31C, BD Pharmingen, BD Biosciences); and mouse IgG1, κappa (clone B11/6) Abcam.

### Human Samples

Human blood samples were collected from fully informed and consenting individuals (written consent was obtained from patients), and human studies were approved by the Jagiellonian University Institutional Bioethics Committee (#87/B/2014; 1072.6120.7.2018; 1072.6120.30.2020; 1072.6120.355.2020). In total, 5 EGPA patients (age 53.4 ± 16.2; M:F 3:2), 12 AD patients (age 37.8 ± 13.1; M:F 6:6), and 88 healthy individuals (age 34.9 ± 9.1; M: F, 82:6) were enrolled in the study. Patients with EGPA fulfilled the 1990 American College of Rheumatology (ACR) Classification Criteria for EGPA (15, 16). Enrollment occurred at the time of diagnosis or any time during the course of their disease (flare or remission). Disease relapse was based on physician-expert clinical judgment and defined as an increase in the activity of the disease. EGPA activity was attested by an increase in the Birmingham Vasculitis Activity Score (BVAS), requiring a dose increase, imitation, or reinstitution of glucocorticoids (GCs) and/or any immunosuppressive drug (17). All but one patient at the time of enrolment was characterized by absolute eosinophil counts (AEC) >1,500/μl. None of the patients at the time of enrollment was treated with strong immunosuppressive drugs (i.e., rituximab, cyclophosphamide, mycophenolate mofetil). The daily dose of GCs was ≤8 mg methylprednisolone. AD patients were diagnosed according to the criteria defined by Hanifin and Rajka (18). Disease severity in AD was measured according to the Severity Scoring of Atopic Dermatitis (SCORAD) and ranged from 26.5 to 83 (50.2 ± 15.2 the mean ± SD). Healthy control subjects had no clinical signs of dermatologic or allergic diseases.

### Isolation of Granulocytes, Eosinophils, and PBMCs

Blood was collected into sodium citrate collection tubes and was subjected to isolation of granulocyte and PBMC fractions within 1 h of draw. PBMCs and granulocytes were isolated using a density gradient 1.077 g/ml centrifugation, with the aid of Pancoll (PAN Biotech). Granulocytes were recovered from the corresponding erythrocyte fraction of the Pancoll gradient as previously described (19). Eosinophils were further purified *via* negative magnetic sorting using the Human Eosinophil Isolation Kit (Miltenyi Biotec, Bergisch Gladbach, Germany) according to the manufacturer’s recommendations. The purity of the isolated cells was examined by flow cytometry based on FSC and SSC parameters and low CD16 immunoreactivity. The eosinophil preparations were routinely ≥94% pure.

### Flow Cytometry

Granulocytes and PBMCs were stained with the following directly conjugated monoclonal mouse anti-human antibodies: anti-CD16 (APC-Cy7, clone: 3G8, BioLegend), anti-CD15 (BV421, clone: W63D, BioLegend), anti-CD14 (PerCP-Cy5.5, clone: MΦP9, BD Pharmingen, BD Biosciences), anti-CD3 (APC, clone: UCHT1, eBioscience), anti-CD19 (BV421, clone: HIB19, BD Horizon, BD Biosciences), anti-CD56 (BV421, clone: NCAM16.2, BD Horizon, BD Biosciences), and anti-CD45 (BV510, clone: HI30, BioLegend). For SLPI staining, granulocytes and PBMCs were first fixed with 3.7% formaldehyde and permeabilized with 0.1% saponin in PBS. Then, unless it is stated otherwise, cells were incubated with primary antibodies—monoclonal-biotin-mouse-anti-human SLPI (clone: 31) or biotin mouse IgG1 к isotype control (clone: MOPC-31C) followed by PE−streptavidin (BD Pharmingen, BD Biosciences). Where indicated, cells were incubated with monoclonal mouse-anti-human SLPI (Creative Diagnostics or R&D clone 20409), or isotype control (mouse IgG1, κappa, clone B11/6), followed by APC-conjugated polyclonal goat anti-mouse IgG1 (Abcam). Flow cytometry was performed on LSRII (BD Biosciences), and the data were analyzed using the software DIVA and FCS Express.

To assess cell viability, granulocytes from healthy donors (100 μl) were preincubated with 20 μg/ml of SLPI for 15 min, in media supplemented with 10% FCS, and then incubated for 2 h at 37°C (0.5 ml; 0.5 × 10^6^ cells/ml). Viability was determined using Annexin V FITC (BD Pharmingen) staining, followed by staining with CD16 mAbs and propidium iodide (PI, BD Pharmingen). Live cells were calculated as Annexin V^-^ and PI^-^.

### Immunocytochemistry

Purified granulocytes or eosinophils were seeded on Permanox plastic slides using the Nunc Lab-Tek Chamber Slide System (Thermo Fisher Scientific) or on poly-L-lysine-coated glass slides (0.8–2 × 10^5^ cells per coverslip) and incubated at 37^◦^C, 5% CO_2_, for 20 min in serum-free RPMI (RPMI 1640, Biowest). Cells were seeded on poly-L-lysine slides and firmly attached to the glass by a gentle centrifugal force (4.5g, 2 min) using a cytospin centrifuge (Cytospin 3, Shandon). Alternatively, immediately after isolation cells were deposited on microscope slides (18g, 10 min) using a cytospin cytocentrifuge.

Cells were then fixed with 3.7% formaldehyde, blocked overnight with 5% FBS, 1% BSA, 0.05% Tween 20, 2 mM EDTA in PBS at 4°C, treated with 0.1% saponin in PBS for 30 min at room temperature, and stained for SLPI using monoclonal-biotin-mouse-anti-human SLPI (clone: 31, Abcam) or biotin mouse IgG1 к isotype control (clone: MOPC-31C, BD Pharmingen, BD Biosciences), followed by PE−streptavidin (BD Pharmingen, BD Biosciences), and counterstained with Hoechst 33258 (Invitrogen) to visualize DNA (14).

Frozen 6–10-μm sections were prepared from skin biopsies of the AD patients, fixed in acetone, and stained with the anti-SLPI Abs or isotype control as above, and with FITC-labeled anti-CCR3 Abs (clone: 5E8, BioLegend) or FITC mouse IgG2b к isotype control (clone: MCP-11, BioLegend). The sections were counterstained with Hoechst 33258. Slides were mounted using Fluorescence Mounting Medium (Dako). Images were captured with a fully motorized fluorescence microscope (Nikon, Eclipse) and analyzed using the software NIS-Elements software (Nikon).

### TEM

Granulocytes and/or sorted eosinophils (1.5 × 10^6^) were fixed overnight in 2.5% glutaraldehyde in 0.1 M sodium cacodylate buffer at 4°C. Cells were then postfixed in 1% osmium tetroxide in 0.1 M cacodylate buffer for 1 h at room temperature and were then stained *en bloc* with 2% uranyl acetate aqueous solution for 1 h at room temperature. Samples were then embedded in epoxy resin (Poly/Bed 812; Polysciences, Inc., Warrington, PA, USA) after dehydration in graded ethanol series (50%–100%) and in propylene oxide. Ultrathin sections (65 nm) were cut using ultramicrotome (Leica EM UC7) and post-stained with uranyl acetate and lead citrate. The specimens were observed using a transmission electron microscope (JEOL JEM-2100) operating at an accelerating voltage of 80 kV.

### Immunogold Labeling

Ultrathin sections of samples placed on nickel grids were incubated with 4% sodium metaperiodate for 10 min and washed in distilled water, followed by 1% aqueous periodic acid for 10 min and blocked in 1% fish skin gelatin (FSG) in PBS for 1.5 h. Sections were incubated with primary biotinylated mouse anti-human SLPI mAbs [IgG1, clone 31 (Abcam)] or biotin mouse IgG1 к isotype control (clone: MOPC-31C, BD Pharmingen, BD Biosciences), in 1% FSG/PBS overnight at 4°C, followed by a secondary antibody (monoclonal mouse anti-biotin IgG1 к, clone 3D6.6, Jackson ImmunoResearch) overnight at 4°C and a tertiary antibody (12 nm Colloidal Gold AffiniPure Donkey Anti-Mouse IgG, Jackson ImmunoResearch) for 2 h at room temperature. Sections were then fixed in 1% glutaraldehyde in PBS and examined in TEM.

### ELISA

Levels of SLPI were quantified in lysates of granulocytes, eosinophils, and plasma using a sandwich ELISA. 1 × 10^6^ cells were lysed in 150 μl RIPA lysis buffer (Sigma) with protease inhibitor cocktail (Roche). Lysates were centrifuged at 16,000 × g for 20 min to remove cells and cellular debris. ELISA stripes (MaxiSorp loose Nunc-Immuno Module, Thermo Scientific) were coated with mouse 5 μg/ml anti-human SLPI mAbs (clone: 20409, Creative Diagnostics) in PBS, overnight at 4°C. For cell lysates, stripes were also coated using monoclonal mouse IgG1 (ICIGG1) isotype control (B11/6, Abcam). The stripes were then washed with PBS with 0.05% Tween 20 and blocked with 3% biotin-free BSA (Roth) for 2 h at room temperature. Samples diluted in blocking buffer were added and incubated at room temperature for 2 h. Recombinant human SLPI was used as a standard. Bound SLPI was detected using biotinylated monoclonal mouse anti-human SLPI antibody (clone: 31, Abcam) followed by incubation with HRP-conjugated streptavidin (BD Pharmingen, BD Biosciences). The absorbance was measured at 450 and 630 nm (as a reference wavelength) using a Tecan Infinite M200 Plate Reader. The absorbance values for isotype controls were subtracted from the total values in each lysate sample and normalized on the basis of total protein levels determined using BCA assay (Sigma-Aldrich).

### Chemotaxis Assay

Granulocytes from healthy donors were preincubated with 20 μg/ml of SLPI for 15 min, in media supplemented with 10% FCS, followed by *in vitro* Transwell chemotaxis assay. 100 μl of cells (3 × 10^5^ cells/well) in RPMI with 10% FCS (Gibco) was added to the top well of 3-μm-pore transwell inserts (Costar or Thermo Fisher), and media or media plus chemoattractant 50 ng/ml CCL11 was added to the bottom well at a volume of 600 μl volume. Migration was assayed for 1.5 h at 37°C. The inserts were then removed, and cells that had migrated through the filter to the lower chamber were collected, stained with anti-CD45, anti-CD16, and anti-CD15 mAbs, and counted using flow cytometry.

### Time-Lapse Video Microscopy

Magnetically sorted eosinophils from healthy donors were seeded on microscopy plates (black uncoated μ-Plate 96-well, ibidi, Munich, Germany) and incubated for 15 min in RPMI supplemented with 10% FCS, or RPMI + 10% FCS and 20 μg/ml SLPI, followed by gentle spinning at 50g for 3 min. The cells were then imaged in Nikon Eclipse with a motorized stage, at ×10 magnification and at a rate of one frame/16 s for 30 min at room temperature. The files generated were analyzed using NIS-Elements software (Nikon). The migrating cells were recognized as those with an elongated shape that adhered to the surface and which displayed any movement in the above time frame. The cells were counted manually and calculated as a percentage of all cells within the fields of view (FOV) in a blinded fashion by two independent investigators.

### Statistical Analysis

Significance was evaluated using the software OriginLab (OriginLab Corporation, MA, USA), and statistical differences were determined using the ANOVA and Mann–Whitney or *t*-test. A *p* value at *p < 0.05, **p < 0.01, or ***p < 0.001 was considered statistically significant.

## Results

### Circulating and Tissue-Recruited Eosinophils Are Immunoreactive for SLPI

**Figure 1:**
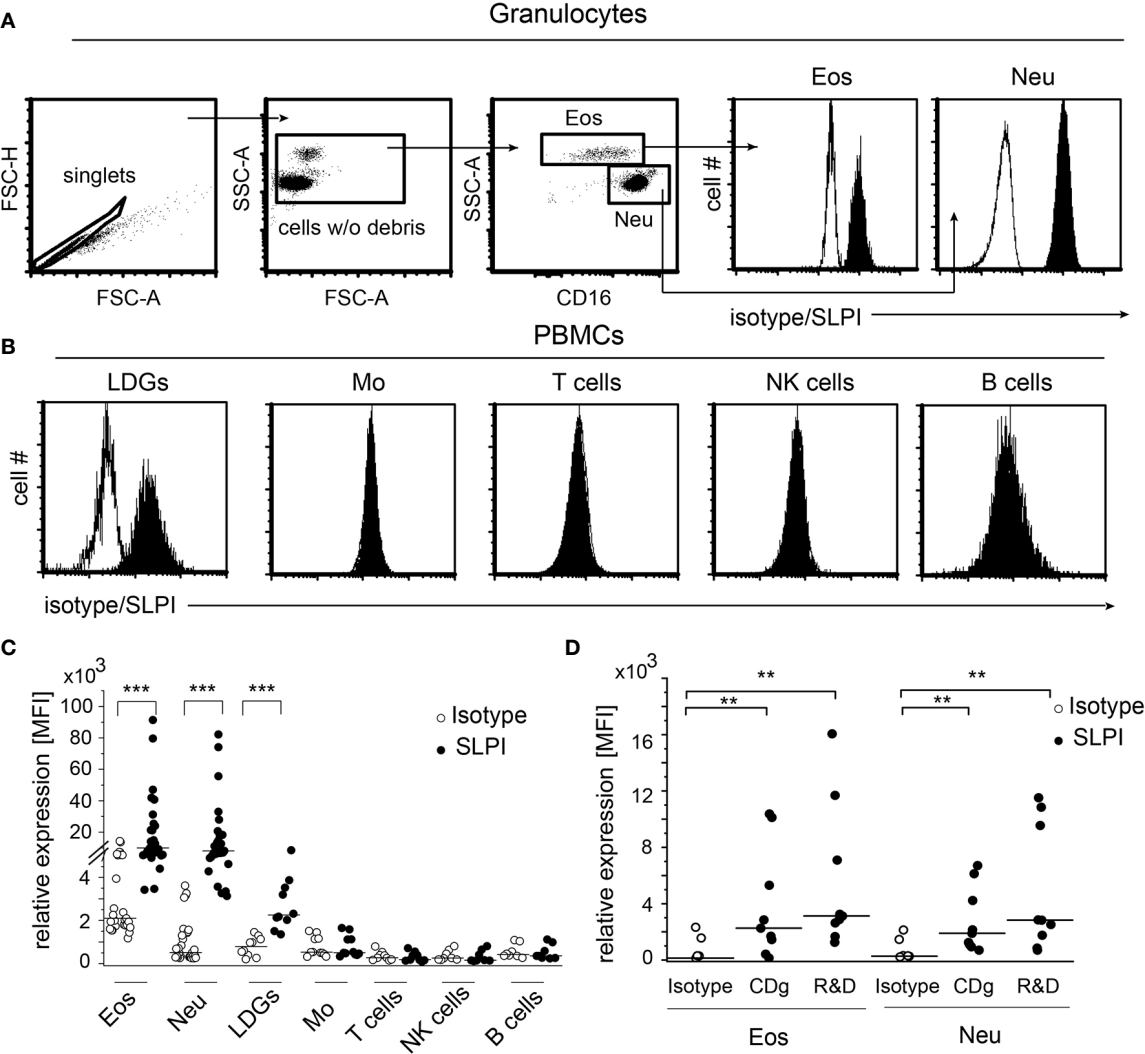
Granulocytes, including eosinophils, are the main source of SLPI among circulating leukocytes in healthy individuals. Granulocytes **(A)** and PBMCs **(B)** from healthy donors were subjected to flow cytometry analysis for SLPI expression using anti-SLPI mAbs (clone: 31, Abcam) (shaded histograms) or isotype control (empty histograms). Eosinophils (Eos) and neutrophils (Neu) were identified in granulocyte fraction on the basis of the gating strategy indicated (dot plots, **A**), whereas LDGs, monocytes (Mo), NK cells, T cells, and B cells in the PBMC fraction were identified on the basis of gating on singlets, cell w/o debris, and staining for CD14, CD3, CD15, CD19, CD56, and/or CD16. Representative histograms for these cells are shown in **(A, B)** Data from all donors are shown as mean fluorescence intensity (MFI), **(C)**. Data from donors stained with alternative anti-SLPI mAbs [Creative Diagnostics (CDg) and R&D] or isotype control are shown for Eos and Neu as MFI **(D)**. Lines indicate the median value for each data set. n = 29 or n = 10 for granulocytes and PBMCs, respectively **(C)** or n = 8 **(D)**. **p < 0.01; ***p < 0.001; Mann–Whitney test.

To determine the cellular sources of SLPI in human blood, we analyzed the SLPI levels in granulocytes and peripheral blood mononuclear cells (PBMCs) isolated from healthy donors using flow cytometry and anti-SLPI or isotype control Abs. The detection of eosinophils in the granulocyte fraction was based on high granularity revealed by the high side scatter parameter (SSC), and low/negative CD16 surface expression, whereas neutrophils were identified on the basis of lower SSC and CD16-positive staining **Figure 1A**). This strategy discriminates effectively between eosinophils (96.9% of CCR3+ cells) and neutrophils (99.9% of CCR3- cells) (**Supplementary Figure 1**). Both neutrophils and eosinophils were found to be strongly positive for SLPI (**Figures 1A, C**). Among PBMCs, we found only low-density granulocytes (LDGs) identified as CD15+, CD14- cells (20), but not monocytes (CD14+, CD3-), T cells (CD14-, CD3+), NK cells (CD14-, CD56+ CD3-, or CD56+ CD16+) or B cells (CD19+, CD3-) to be immunoreactive for SLPI (**Figures 1B, C**). Moreover, significant immunostaining for SLPI in both eosinophils and neutrophils was observed using three different anti-SLPI mAbs (**Figures 1A, C, D**).

**Figure 2:**
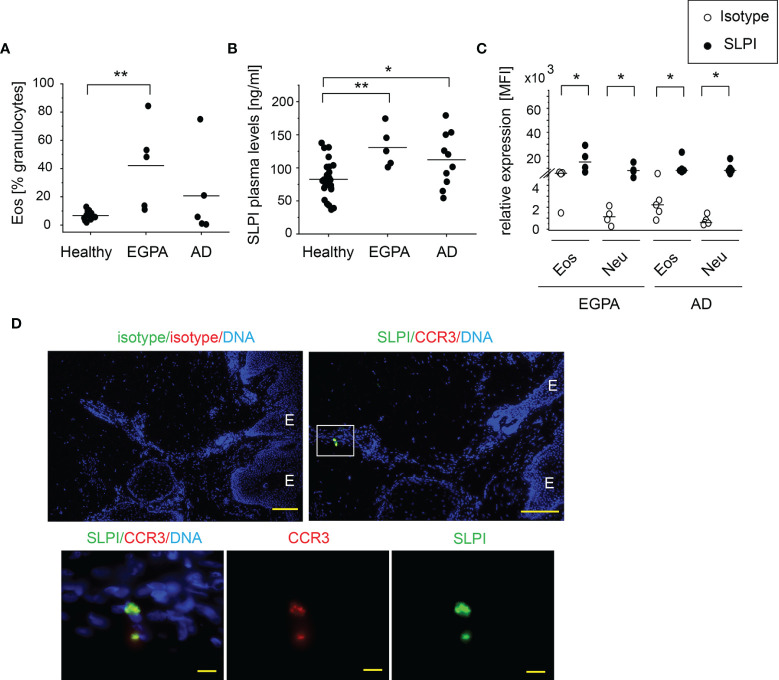
Patients with blood eosinophilia demonstrate high levels of SLPI in plasma and SLPI-immunoreactive granulocytes. The donors indicated were subjected to isolation of granulocytes, plasma, and/or skin biopsies. Dots indicate individual donors and lines the mean or median value for each data set. **(A)** % of eosinophils among granulocytes was determined using flow cytometry. Lines indicate the mean, n = 5–13, **p < 0.001; ANOVA, Tukey *post hoc*. **(B)** SLPI levels in plasma were quantified using ELISA. Lines indicate the mean, n = 5–26, **p < 0.01; *p < 0.05; Tukey ANOVA. **(C)** Levels of SLPI were determined using flow cytometry. Lines indicate the median, n = 4–5, *p < 0.05; Mann–Whitney test. **(D)** Fluorescence microscopy images of affected skin of AD patient. Skin was stained for SLPI or isotype control (green), CCR3 to detect eosinophils, or isotype control (red) with Hoechst counterstain to detect DNA (blue). An overlay and single-channel images from one donor. Data are representative for 3 donors. E indicates epidermis. Scale bar = 50 μm, or 10 μm (enlarged images).

An increased number of circulating eosinophils are associated with EGPA and AD. As anticipated, EGPA and AD patients presented with a significantly higher and a tendency to a higher percentage of eosinophils among circulating granulocytes, respectively (**Figure 2A**). These conditions were also found to manifest in significantly elevated SLPI levels in plasma (**Figure 2B**). Flow cytometry analysis showed that in common with healthy individuals, eosinophils and neutrophils from EGPA and AD patients express high levels of SLPI (**Figure 2C**). Moreover, eosinophils that infiltrate the affected skin of patients with AD were positive for SLPI (**Figure 2D**). Together, these data further point to a possible association between eosinophils and SLPI in these disorders.

### Staining for SLPI Does Not Reflect Total SLPI Levels in Eosinophils

**Figure 3:**
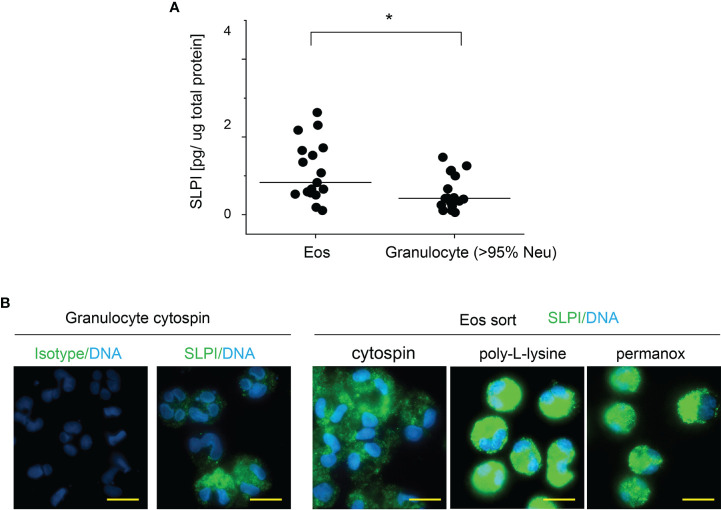
Eosinophils have higher SLPI protein levels compared to neutrophils, but their immunoreactivity for SLPI varies with experimental conditions. Granulocytes and eosinophils were isolated from the blood of healthy donors. **(A)** The cells indicated were subjected to cell lysis followed by SLPI-specific ELISA. Data are normalized to total protein levels determined by BCA. Dots indicate individual donors and lines the median value for each data set. n = 17, *p < 0.05; Mann–Whitney test. **(B)** The cells were directly deposited onto microscope slides *via* centrifugation (cytospin) or were first incubated on poly-L-lysine-covered slides for 20 min followed by short cytospin (poly-L-lysine) or were incubated on Permanox slides for 20 min (Permanox). The cells were then stained using biotinylated mouse anti-human SLPI mAbs or isotype control mAbs followed by PE-labeled streptavidin. Data are from one donor and are representative of at least 2 donors. Scale bar = 10 μm.

**Figure 4:**
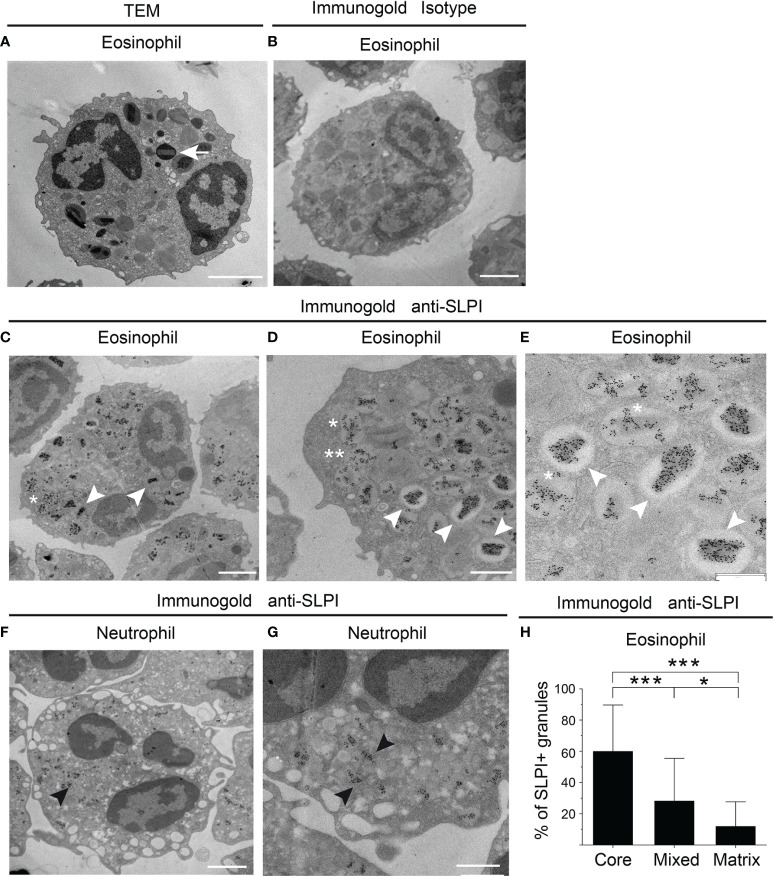
SLPI mainly localizes to eosinophil crystalline cores. The granulocytes indicated from healthy donors were subjected to conventional TEM **(A)** or immuno-electron microscopy **(B–H)** using biotinylated mouse anti-human SLPI mAbs or isotype control mAbs followed by mouse anti-biotin mAbs and gold-labeled donkey anti-mouse Abs. **(A)** The ultrastructure of eosinophil is depicted by TEM with characteristic crystalline core in the granule (arrow). **(B)** Isotype control for SLPI staining in eosinophils. **(C–E)** Localization of SLPI to crystalline core in eosinophils (white arrowheads), crystalline core and matrix (mixed localization, asterisk), or granule matrix (double asterisks), revealed by immunogold staining for SLPI and shown at lower- and higher-power magnification. **(F, G)** Immunogold localization of SLPI in neutrophils shown at lower- and higher-power magnification (black arrowheads). **(H)** Average distribution per single cell of granules with the indicated SLPI localization in electron micrographs. The mean ± SD, n = 17 eosinophils from two donors is shown, *p < 0.05, ***p < 0.001 by ANOVA, with *post hoc* Tukey test. Scale bar = 2 μm **(A–C, F)**, 1 μm **(D, G)**, or 0.6 μm **(E)**.

Flow cytometry analysis of SLPI levels in granulocytes suggested that eosinophils contain less SLPI compared to neutrophils (**Figures 1A, C**). However, ELISA revealed that the lysates of purified eosinophils derived from healthy individuals contain different, but overall significantly higher, levels of SLPI protein compared to neutrophils (**Figure 3A**). Likewise, immuno-fluorescence microscopic examination of granulocytes from healthy donors showed overall stronger staining of eosinophils for SLPI compared to neutrophils from the same donors (**Figure 3B**). Moreover, fluorescence microscopy of the sorted eosinophils from the same donors demonstrated different levels of immunostaining for SLPI in cells that were immediately attached to microscope slides using a cytocentrifuge or were first cultured on poly-L-lysine for 20 min followed by a short cytospin or were cultured on a Permanox plastic surface in chamber slides for 20 min (**Figure 3B**). Together, these data suggested that eosinophils express more SLPI compared to neutrophils but demonstrate assay-dependent immunostaining for SLPI. The different immunoreactivity could result from condition-dependent subcellular localization and/or interaction with other molecules. Ultrastructural immunogold analysis using TEM localized SLPI mainly to the crystalline core compartment of the eosinophil granules (**Figures 4A–E, H**). Neutrophils, devoid of crystalline core (21), stored SLPI in granules, but in what appeared to be a less-compacted manner (**Figures 4F, G**). In addition, immuno-electron microscopy of eosinophils showed that in some granules SLPI is not restricted to the electron-dense crystalline core but is also detected in what appears to be an electron-lucent matrix (**Figures 4D, E, H**). We conclude that SLPI is present in partly different subcellular localizations in eosinophils and neutrophils.

### Exogenous SLPI Regulates Numbers of Migratory Eosinophils and Their CCL11-Driven Chemotaxis

High levels of SLPI in eosinophils suggest that this protein may be involved in the regulation of eosinophil functions, such as viability or migration. However, preincubation of eosinophils with 20 μg/ml of SLPI followed by 2-h incubation did not significantly affect the lifespan of eosinophils. 92.8 ± 3.6% and 91.2 ± 5.8% of eosinophils remained viable when cultured in the absence or presence of SLPI, respectively (n = 5 donors, viability at t_0_ = 96.2 ± 1.9%).

**Figure 5:**
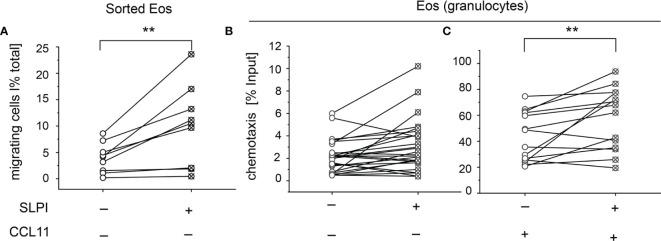
Exogenous SLPI impacts eosinophil migration capacity. The cells indicated were derived from healthy donors and incubated with media supplemented with 10% FCS (-) or media supplemented with 10% FCS and 20 μg/ml SLPI for 15 min followed by migration assays. **(A)** Time-lapse video microscopy showing % of migratory cells in total cells per FOV, n = 9. **p < 0.001; *t*-test. **(B)** Transwell chemotaxis assay to media only, n = 17; **(C)** Transwell chemotaxis assay to 50 ng/ml CCL11, n = 10. Symbols indicate individual donors and lines connect data sets from the same individuals. **p < 0.001; Mann–Whitney test.

Eosinophils are recruited into various peripheral locations under homeostatic, infectious, and inflammatory conditions. To determine whether SLPI regulates eosinophil migration, we incubated eosinophils from the peripheral blood of healthy donors with recombinant human SLPI in media supplemented with 10% FCS, or media with 10% FCS as a control, followed by two migration assays: time-lapse video microscopy and Transwell chemotaxis assay. Since media supplemented with 10% FCS contained approximately 4 mg/ml of the total protein by BCA assay, the addition of 20 μg/ml SLPI did not significantly change the total protein levels (protein levels were on average 4 ± 0.7 vs. 3.9 ± 0.5, mean ± SD, for samples w/o and with SLPI, respectively, n = 3). Using time-lapse video microscopy, we first analyzed the ability of SLPI to regulate the migration of purified, magnetically sorted eosinophils, by manually scoring the number of migrating cells. Video microscopy performed for 30 min demonstrated that a significantly higher number of eosinophils exhibited migratory behavior when they were pretreated with SLPI for 15 min prior to seeding in a tissue culture dish and video recording (**Figure 5A** and **Supplementary Video 1**). Likewise, when the functional migratory responses of SLPI-treated or untreated granulocytes were analyzed using Transwell chemotaxis assay, we observed a similar tendency for a higher number of migrating eosinophils in the presence of SLPI at the base line (**Figure 5B**). In addition, eosinophils in granulocyte fractions incubated with SLPI were more efficient in their chemotactic response to eosinophil-specific CCL11 (**Figure 5C**). Together, these data suggest that SLPI regulates the migratory potential of eosinophils.

## Discussion

In this study, we report previously unknown links between human eosinophils and SLPI of relevance to eosinophil-mediated defense responses and allergy-based inflammatory disorders. Although SLPI has been shown to act as an anti-inflammatory molecule in myeloid cells such as macrophages and DCs (12), the role of this protein in granulocytes is much less defined. As far as human eosinophils are concerned, there are virtually no reports. This is surprising in the light of our data that show strong immunoreactivity for SLPI in human blood eosinophils and in eosinophils that infiltrate the disease-altered skin of AD patients (**Figures 1**, **2**). Although immunostaining for intracellular SLPI in granulocytes is subject to condition-dependent variability, and thus does not unequivocally reflect the total levels of this protein either in neutrophils (20) or in eosinophils (**Figure 3**), the latter appear to be a primary source of SLPI among circulating granulocytes. This conclusion is based on higher SLPI levels in lysed eosinophils compared to neutrophils (**Figure 3**), where potential restraints caused by, for example, epitope masking, are likely removed following cell lysis.

One of the signature features of eosinophils is the presence of crystalline core in their granules. Immuno-electron microscopy showed that SLPI mainly localized to crystalline cores in eosinophils but was also found in the granule matrix (**Figure 4**). Since activation of eosinophils is associated with structural changes within granules, including content emptying (22), condition-dependent alterations, such as cell adhesion vs. cell suspension, could affect SLPI localization in eosinophils and/or the form of arrangement in the crystalline cores. Reorganization of core contents and the appearance of SLPI in other cellular localizations could result in better accessibility of SLPI for detection with anti-SLPI Abs in eosinophils.

In healthy individuals, SLPI protein levels in eosinophil lysates and plasma varied several-fold (**Figures 2**, **3**, respectively), possibly reflecting the different functional status of eosinophils, including degranulation. Alternatively, given that increased SLPI concentration in plasma is associated with allergic inflammatory responses (**Figure 2**), high SLPI levels in eosinophils and/or the blood may mark Th2-biased healthy individuals. Nevertheless, the presence of SLPI in eosinophils from every donor suggests that SLPI is important for the intrinsic or external functions of eosinophils.

Staining for SLPI in eosinophils derived from healthy and patient donors appeared to be similar (**Figures 1C**, **2C**, respectively), suggesting that SLPI levels and/or accessibility for Abs do not differ between healthy individuals and individuals with EGPA or AD. However, both EGPA and AD patients had overall higher eosinophil numbers in the blood and demonstrated significantly increased SLPI plasma levels compared to healthy individuals (**Figures 2A, B**). Together, these data suggest that SLPI-upregulated levels in the blood of EGPA and AD patients can be potentially traced to eosinophilia (**Figure 2**). However, we also acknowledge that elevated SLPI levels in patients could arise from other sources, such as neutrophils, epithelial cells, or platelets (12).

Our previous studies implicated SLPI in the regulation of the release of neutrophil extracellular traps (NETs) in neutrophils. SLPI, an antagonist of neutrophil elastase (NE), can restrain NET deposition in neutrophils partially through controlling NE-dependent histone processing (14). Eosinophils contain neither NE nor cathepsin G, another SLPI-controlled neutrophil enzyme (23), suggesting that SLPI inhibits different proteolytic activities inside eosinophils. Alternatively, or in addition to its antiprotease-dependent or independent intracellular role, eosinophil-derived SLPI could modulate cell function following its release from eosinophils. This is supported by the localization of SLPI in eosinophil granules, suggesting that in common with other granule-stored proteins, such as ECP or MBP, SLPI is likely to be discharged into the extracellular milieu through degranulation.

Extracellular SLPI is thought to relatively freely penetrate the cell membrane (12). Although eosinophils appear to be less efficient compared to neutrophils in taking up recombinant human SLPI *in vitro* (data not shown), they did respond to the extracellular recombinant SLPI with changes in numbers of migratory cells. Thus, extracellular SLPI could influence the eosinophil migratory capacity either through acting on the cell surface and potentially specific cell-surface receptors, and/or *via* modifying intracellular pathways after being taken up by eosinophils from the extracellular milieu.

A regulatory role of SLPI in cell migration was reported previously, but with opposite results. On the one hand, an SLPI deficiency was shown to augment eosinophil-mediated airway inflammation in experimental mouse allergy models. This effect was potentially attributed to an increase in eosinophil influx into sites of allergen challenge and higher expression of proinflammatory mediators by eosinophils in the absence of SLPI (13). Likewise, a suppressive effect of topically applied SLPI on eosinophil recruitment in the model of allergic conjunctivitis in guinea pigs was previously shown (24). Together, these studies pointed to SLPI-dependent inhibition of eosinophil infiltration at the sites of inflammation. On the other hand, a recent report demonstrated decreased migration of SLPI-deleted human gingival carcinoma cells compared to their wild-type counterparts (25). Our data on human eosinophils are in line with these recent studies and suggest that SLPI supports the migration of human cells by increasing numbers of migratory eosinophils. In the case of the first two studies referred to, which examined SLPI function *in vivo* or *ex vivo*, the effect of SLPI was likely more complex in these models and not limited to regulating migration attributes in circulating eosinophils by SLPI. For example, SLPI-restricted release/activation of chemotactic factors by proteases might explain the more efficient recruitment of eosinophils to sites of allergen challenge in the absence of SLPI. Alternatively, a different source of eosinophils, mice, or guinea pigs in the case of these reports, versus human blood in our case, might contribute to these differences. A potential species-dependent effect of SLPI on cell migration might be consistent with the SLPI-facilitated migration of human gingival carcinoma cells (25).

Migration into tissues directed by chemotactic factors is critical for the defensive functions of leukocytes. SLPI exerts important control on human eosinophils by increasing numbers of migratory cells and their directional migration in response to chemokine CCL11 (**Figure 5**). Since CCL11 plays a key role in eosinophil recruitment into sites of inflammation, potential integrative elevation of eosinophil and SLPI levels in EGPA and AD might at least in part account for more robust eosinophil tissue infiltrates, and consequently the heightened eosinophil-mediated tissue changes.

## Data Availability Statement

The data that support the findings of this study are available from the corresponding author upon reasonable request.

## Ethics Statement

The studies involving human participants were reviewed and approved by the Jagiellonian University Institutional Bioethics Committee. The patients/participants provided their written informed consent to participate in this study.

## Author Contributions

JS-M, EP, and JC conceived and designed the experiments. OO, JS-M, AgM, AnM, and BB performed and analyzed the experiments. PM, MK-M, JK-W, and MP contributed the reagents and materials. JC wrote the manuscript and acquired funding. All authors contributed to the article and approved the submitted version.

## Funding

This research was funded by the Polish National Science Center (NCN), grant number UMO-2017/25/B/NZ6/01003.

## Conflict of Interest

The authors declare that the research was conducted in the absence of any commercial or financial relationships that could be construed as a potential conflict of interest.

## Publisher’s Note

All claims expressed in this article are solely those of the authors and do not necessarily represent those of their affiliated organizations, or those of the publisher, the editors and the reviewers. Any product that may be evaluated in this article, or claim that may be made by its manufacturer, is not guaranteed or endorsed by the publisher.
