# Supplementary material for: Secretory Leukocyte Protease Inhibitor Is Present in Circulating and Tissue-Recruited Human Eosinophils and Regulates Their Migratory Function
Source: Front Immunol. 2022 Jan 12;12:737231. doi: 10.3389/fimmu.2021.737231 (PMC8789888; doi:10.3389/fimmu.2021.737231)
Supplement: Supplementary file 1 [file DataSheet_1.pdf]

Supplemetary Figure 1

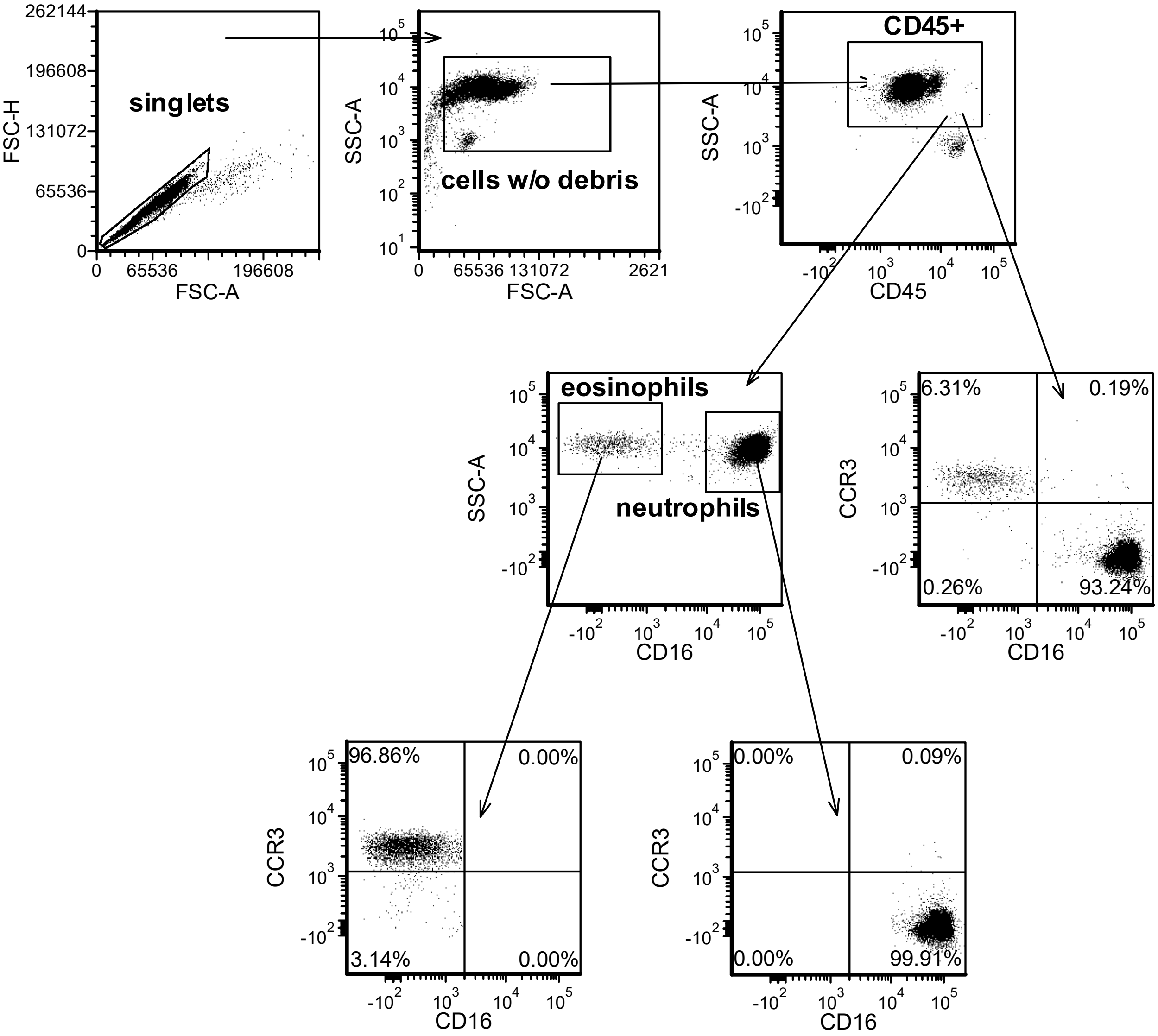

Granulocytes were stained with the following directly-conjugated monoclonal mouse anti-human antibodies: FITC conjugated anti-CCR3 (clone: 5E8, BioLegend) or FITC conjugated mouse IgG2b κ isotype control (clone: MPC-11, BioLegend), APC-Cy7 conjugated anti-CD16 (clone: 3G8, BioLegend) and BV510 conjugted anti-CD45 (clone: HI30, BioLegend). Cells were fixed with 3.7% formaldehyde and analyzed using flow cytometry. Samples were acquired on LSRII (BD Biosciences) and the data were analyzed using the software DIVA and FCS Express.
